# Characterization of the structural, oxidative, and immunological features of testis tissue from Zucker diabetic fatty rats

**DOI:** 10.1515/biol-2022-0495

**Published:** 2022-11-07

**Authors:** Eva Tvrdá, Ján Kováč, Filip Benko, Michal Ďuračka, Anikó Varga, Oľga Uličná, Viera Almášiová, Marcela Capcarová, Mária Chomová

**Affiliations:** Institute of Applied Biology, Faculty of Biotechnology and Food Sciences, Slovak University of Agriculture in Nitra, Tr. A. Hlinku 2, 949 76 Nitra, Slovakia; Third Intern Clinic, Comenius University in Bratislava, Bratislava, Slovakia; Department of Morphological Disciplines, University of Veterinary Medicine and Pharmacy in Košice, Košice, Slovakia; Institute of Medical Chemistry and Clinical Biochemistry, Comenius University in Bratislava, Bratislava, Slovakia

**Keywords:** diabetes mellitus type 2, obesity, testes, oxidative stress, inflammation

## Abstract

The purpose of this study was to characterize the testicular profile of Zucker diabetic fatty (ZDF) rats presenting with type 2 diabetes mellitus (DM2) in the absence or presence of obesity. To achieve this, testes were collected from 270-day-old male Wistar (*n* = 15), ZDF nonobese (*n* = 15), and ZDF obese rats (*n* = 16). Changes to the testicular structure were quantified morphometrically, while immunocytochemistry was employed to assess caspase-3 activity. Reactive oxygen species (ROS) production, fluctuations of major antioxidant molecules, and the extent of damage to the proteins and lipids were assessed in tissue lysates. Levels of selected interleukins (ILs) were determined by enzyme-linked immunosorbent assay. The results reveal significant alterations to the testicular structure accompanied by caspase-3 overexpression, particularly in ZDF obese rats. The most notable disruption of the oxidative balance, characterized by ROS overproduction, antioxidant deficiency, protein, and lipid deterioration was recorded in ZDF rats suffering from both DM2 and obesity. Accordingly, the highest concentrations of pro-inflammatory IL-1, IL-6, and IL-18 accompanied by reduced levels of the anti-inflammatory IL-10 were found in testicular tissue collected from ZDF obese rats. This study highlights the vulnerability of male gonads to pathophysiological changes caused by hyperglycemia, which are further exacerbated by excessive adipose tissue.

## 1 Introduction

Diabetes mellitus (DM) comprises a group of metabolic ailments characterized by hyperglycemia arising from alterations in the secretion and/or activity of insulin. The resulting chronic high blood sugar levels may contribute to the dysfunction of numerous organs, particularly the kidneys, eyes, nerves, heart, and blood vessels [1,2]. The underlying cause for DM-inflicted damage to the metabolism of carbohydrates, lipids, and proteins lies in an insufficient action of insulin on target tissues. This phenomenon is triggered by either inadequate insulin secretion or a decreased response of cellular targets to insulin at one or more points of the endocrine network. A decreased insulin secretion and activity often coexist in one patient; hence, it may be difficult to determine which of these abnormalities is directly responsible for the development of hyperglycemia [2]. DM may be divided into three types: DM type 1 (DM1), DM type 2 (DM2), and DM type 3 (DM3) – also known as gestational DM. DM1 is triggered by autoimmune destruction of pancreatic beta cells (β-cells) and is generally diagnosed in children or adolescents [1,2].

According to Reed et al. [3], DM2 is nowadays the most prevailing metabolic disease, affecting more than 450 million people worldwide. DM2 pathogenesis is characterized by abnormalities in glucose and lipid metabolism, including insufficient insulin secretion by the β-cells and insulin resistance. Target cells are then unable to absorb glucose, leading to the occurrence of hyperglycemia [1,3]. Chronically elevated concentrations of blood sugar may subsequently promote the accumulation of polyols, oxidative insults to healthy tissues, chronic inflammation, and cell death through apoptosis or necrosis [3,4,5]. While the exact etiology of DM2 is still not fully understood, it is known that being obese increases the risk of developing the disease. In fact, the recent research suggests that obese people are up to 80 times more likely to present with DM2 than those with a normal body mass index [6]. A frequent phenomenon interlinking DM with obesity is the metabolic syndrome, which refers to a complex of health issues including insulin resistance, hyperinsulinemia, hyperglycemia, hypertension, and hypercholesterolemia [6,7].

Male gonads may be characterized by a unique architecture, organization, and function. A continuously ongoing process of spermatogenesis presents with high energy requirements and an exceptional metabolic activity of male germ cells, which on the other hand renders the testes to be a highly sensitive barometer to any imbalance of the internal milieu or external stimuli. As reviewed by Condorelli et al. [8], DM could have detrimental effects on male fertility, most notably on the semen quality, translated into decreased sperm motion, excessive DNA fragmentation, and abnormal seminal plasma characteristics. Similar to DM, obesity has been acknowledged as a factor substantially contributing to suboptimal male reproductive performance. Fertility among obese subjects may be affected by sexual dysfunction, endocrinopathy, aromatization activity, thermal stress, as well as inflammatory and obstructive elements of testicular and epididymal pathology [9]. While DM2 and obesity share a mutual relationship, their precise mechanism of action on the male reproductive system needs further understanding.

Animal models play a pivotal role in monitoring and understanding DM pathogenesis due to a combination of their genetic and functional characteristics. Nevertheless, most studies take advantage of rodents subjected to streptozotocin (STZ) that causes pancreatic β-cell destruction and is widely used to induce symptoms consistent with DM1. In the meantime, metabolic studies by and large employ a high-fat diet-induced obesity in animals, which may or may not develop DM2 [10]. To assess a shared impact of obesity and DM2, Zucker diabetic fatty (ZDF) rats seem to act as an optimal model. ZDF rats present with a mutation in the leptin receptor gene, which causes leptin unable to bind to the saturation center in the brain. The animal suffers from consistent hunger and develops obesity when fed, which is subsequently followed by the occurrence of DM2. The animals are characterized by progressive β-cell dysfunction, glucose intolerance, severe insulin resistance, and hyperlipidemia and become diabetic at 8 weeks of age when fed a diet containing at least 6.5% fat [11]. This profile mimics a progressive loss of glucose-stimulated insulin secretion in humans suffering from DM2, and hence, ZDF rats represent the best laboratory model to study human DM2, its pathophysiology, and the effects of potential therapeutic options [11,12,13].

Despite an array of studies employing male ZDF rats ref. [11], only a few reports have strived to assess their reproductive characteristics. Evidence gathered from these studies indicates a marked testicular atrophy accompanied by the detachment and disorganization of germ cells [14,15,16,17]. Moreover, a global transcriptomic analysis revealed changes in the genes involved in the lipid metabolism [18], which may lead to a diminished fertility in ZDF males. Nevertheless, very little is known about the involvement of oxidative stress and inflammation in the reproductive dysfunction of ZDF rats. As such, our aim is to provide a comprehensive description of the histological, oxidative, and immunological profile of the testicular tissue collected from ZDF rats diagnosed with DM2 in the presence or absence of obesity.

## 2 Materials and methods

### 2.1 Animals

This study employed 46 adult (270 days) male rats, which were obtained from the Institute of Experimental Pharmacology (Slovak Academy of Sciences, Dobrá Voda, Slovakia) and kept in plastic cages at 24 ± 1°C and a 12 h light/12 h dark photoperiod. All animals were provided with drinking water *ad libitum* [19,20].

The rats were divided into three groups: the control group included Wistar rats (*n* = 15), the first experimental group comprised ZDF (fa/fa) nonobese rats (*n* = 15; ZDF nonobese (ZN)), and the second one consisted of ZDF (fa/fa) obese rats (*n* = 16; ZDF obese (ZO)). The control and ZN groups were fed under a controlled regime, while the ZO group had unrestricted access to the chow (Purina Rodent LabDiet 5008, IPS Product Supplies, UK) with a fat content of 6.50%. Fasting blood glucose levels were monitored using a FreeStyle Optium Neo Glucose and Ketone Monitoring System (Abbott Diabetes Care Ltd., UK) with a measurable extent of 1.1–27.8 mmol/L. One blood drop was obtained from the tail vein in the morning between 6:30 and 8:00 A.M. every 2 weeks. Diabetes was acknowledged when the blood glucose level was equal to or higher than 17 mmol/L [19]. By week 8, all ZN and ZO rats had developed hyperglycemia that remained persistent until animal sacrifice.

**Figure 1:**
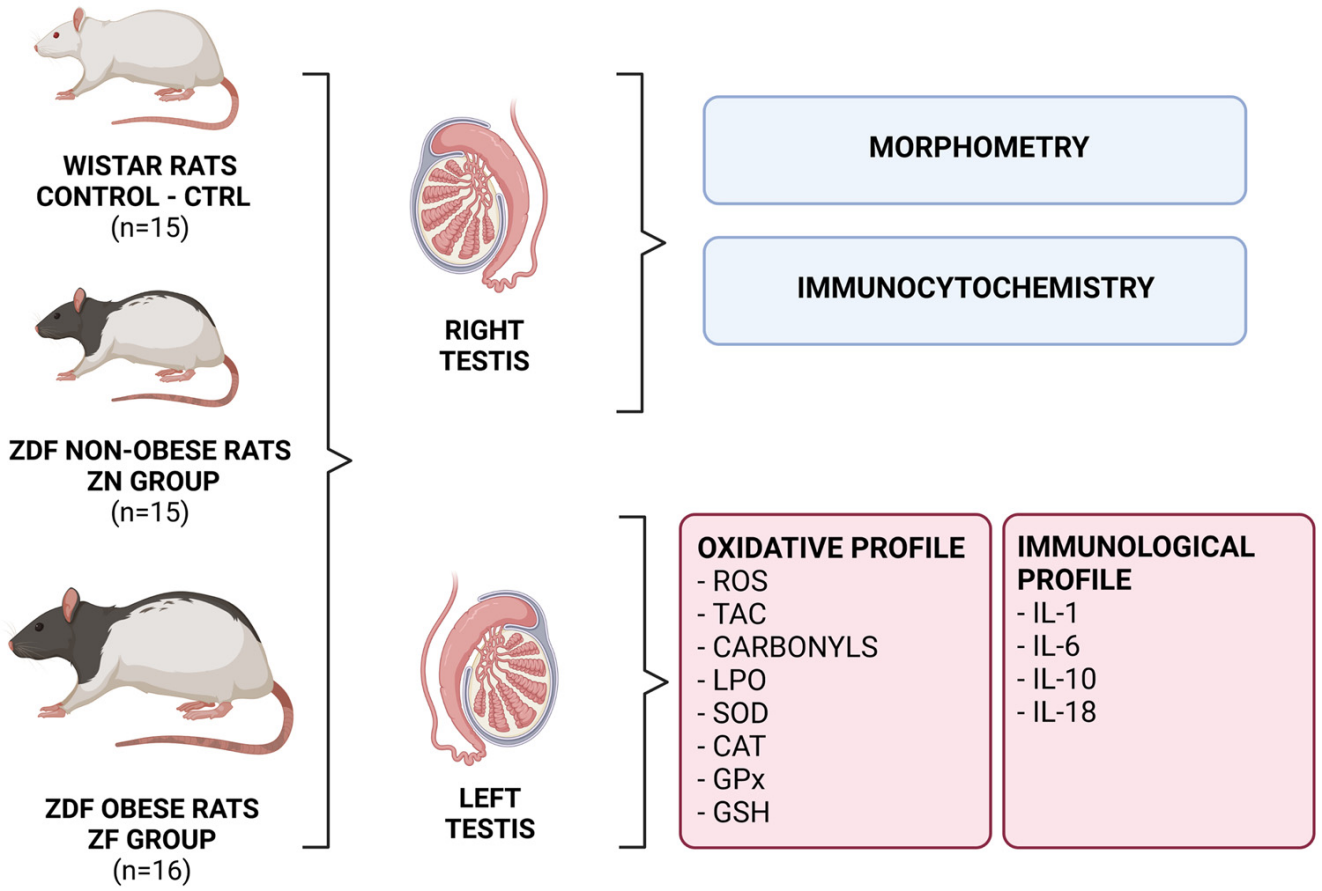
Overview of the experimental approach. ROS – reactive oxygen species, TAC – total antioxidant capacity, LPO – lipid peroxidation, SOD – superoxide dismutase, CAT – catalase, GPx – glutathione peroxidase, GSH – glutathione, IL-1 – interleukin-1, IL-6 – interleukin-6, IL-10 – interleukin-10, IL-18 – interleukin-18. Figure created with BioRender.com.


The final glycemic measurement (Table 1) was performed shortly before animal sacrifice by sevoflurane anesthesia and decapitation. Testes were immediately excised from the scrotum, dissected from the epididymis, and weighted. The right testes were processed for histological analyses, while the left testes were used for lysis and subsequent biochemical assays. A resume of the experimental approach is outlined in Figure 1.

**Table 1:** Glycemia and testicular characteristics of the control and experimental groups

	Control (Ctrl; *n* = 15)	ZDF nonobese (ZN; *n* = 15)	ZDF obese (ZO; *n* = 16)
Fasting glucose level (mmol/L)	5.12 ± 0.81	17.59 ± 0.9^***Ctrl^	19.45 ± 0.94^****Ctrl^
Testicular weight (g)	1.75 ± 0.06	1.38 ± 0.05^*Ctrl^	1.50 ± 0.05
**Relative volume of testicular structures**			
Seminal epithelium (%)	72.88 ± 5.55	60.38 ± 8.79^*Ctrl^	50.55 ± 7.14^***Ctrl,*ZN^
Lumen (%)	23.05 ± 4.88	36.20 ± 3.97^*Ctrl^	44.02 ± 5.41^**Ctrl^
Interstitium (%)	4.07 ± 1.01	3.42 ± 0.87	5.43 ± 1.10
**Morphometric characteristics of the seminiferous tubules**			
Tubular diameter (µm)	226.52 ± 6.42	187.10 ± 7.58^**Ctrl^	166.17 ± 6.24^***Ctrl,*ZN^
Lumen diameter (µm)	162.78 ± 7.47	139.22 ± 6.54^**Ctrl^	122.76 ± 6.79^***Ctrl,*ZN^
Epithelial height (µm)	66.58 ± 5.12	58.22 ± 4.37^*Ctrl^	51.13 ± 5.03^***Ctrl^

Mean ± SD. ^*^ *P* < 0.05; ^**^ *P* < 0.01; ^***^ *P* < 0.001; ^****^ *P* < 0.001. ^Ctrl^ – versus control; ^ZN^ – versus ZDF non-obese.

**Ethical approval:** The research related to animal use has been complied with all the relevant national regulations and institutional policies for the care and use of animals and was approved by the State Veterinary and Food Administration of the Slovak Republic (no. 493/18-221/3) and by the Ethical Committee of the Institute of Medical Chemistry, Biochemistry and Clinical Biochemistry, following the Directive 2010/63/EU of the European Parliament.

### 2.2 Testicular histology and morphometry

Testicular samples were fixed in 10% formol (Centralchem, Bratislava, Slovakia), dehydrated in a grade series of 70, 80, 90, and 100% ethanol (Centralchem, Bratislava, Slovakia), saturated with benzene (Centralchem, Bratislava, Slovakia), and embedded with paraffin (Centralchem, Bratislava, Slovakia). The resulting blocks were sectioned with a microtome into 5 µm thick sections, which were subsequently stained with hematoxylin and eosin (Sigma-Aldrich, St. Louis, MO, USA). Photographs were taken at a magnification of 10× and 20× using the Leica EC3 optical microscope (Leica Camera AG, Wetzlar, Germany). Fine morphometric quantification was performed using the QuickPHOTO MICRO program (Promicra, Prague, Czech Republic). Relative volume (%) of the germinal epithelium, lumen, and interstitium were quantified in the tissue samples. Tubular diameter, luminar diameter, and the height of the epithelium (µm) were measured as well [21].

### 2.3 Immunocytochemistry

For the immunocytochemical assessment of caspase-3 activity, testicular tissue was fixed in 10% formol, dehydrated in ethanol series, embedded with paraffin, and sectioned into 5 µm sections. The resulting sections were deparaffinized in xylene (Sigma-Aldrich) and rehydrated in a graded series of ethanol. Subsequently, the sections were treated with 10 mM citrate buffer (pH 6.0) twice for 5 min each and cooled down. Following washing three times in phosphate buffer (PBS; Sigma-Aldrich), endogenous peroxidases were deactivated by 3% hydrogen peroxide (H_2_O_2_; Sigma-Aldrich) for 20 min, and the sections were washed again three times with PBS. For the blocking procedure, the samples were treated with Animal-Free Blocking Solution (Cell Signaling Technology, Danvers, MA, USA) for 10 min. Subsequently, the samples were incubated at 4°C with the rabbit Caspase 3 primary antibody (#9662, 1:1,000; Cell Signaling Technology) overnight. After washing with PBS, the sections were incubated with an antirabbit, horseradish peroxidase-linked secondary antibody (#7074, 1:1,000; Cell Signaling Technology), washed again in PBS, and visualized with diaminobenzidine (10%; Roche Diagnostics Corporation, IN, USA). The sections were finally mounted with the Permount mounting medium (Fisher Scientific, Hampton, NH, USA) on glass slides. Photographs were taken with the Leica EC3 optical microscope at a primary magnification of 10× and 20× (Leica Camera AG; Wetzlar, Germany) [22,23].

Assessment of the immunocytochemical reaction was performed using the H-SCORE system based on a semi-quantitative analysis distinguishing among the following categories: 0 (no staining), 1+ (weak/faint yellow staining), 2+ (moderate/brownish yellow staining), and 3+ (intense/brown staining). The calculation of the H-SCORE followed the formula previously used by Ayan et al. [22]. Five randomly selected areas per each slide were assessed under a light microscope (10× and 20× primary magnification).

### 2.4 Preparation of tissue lysates

For the biochemical assays, testicular tissue samples of approximately 50 mg were pretreated with RIPA buffer (Sigma-Aldrich) containing a protease inhibitor cocktail (Sigma-Aldrich) and subsequently sonicated on the ice at 28 kHz for 30 s. Following centrifugation (11,828×*g*, 4°C, 15 min), the lysates were collected and stored at −80°C [24].

Before analyses of the oxidative and immunological profile, protein concentration was quantified in each lysate, which was necessary for a posterior data normalization. Each lysate was processed with the total protein commercial kit based on the Biuret method (DiaSys Diagnostic Systems, Holzheim, Germany), and the total amount of proteins was assessed with the help of the Rx Monza semi-automatic analyzer (Randox Laboratories Ltd., Crumlin, UK) [24,25].

### 2.5 Oxidative profile analysis

Assessment of the reactive oxygen species (ROS) production in each sample followed the chemiluminescence assay employing luminol (5-amino-2,3-dihydro-1,4-phthalazinedione; Sigma-Aldrich). The lysates were treated with luminol (2.5 μL, 5 mmol/L), while the negative controls consisted of 100 μL PBS. Positive controls comprised 100 μL PBS, 2.5 μL luminol, and 12.5 μL H_2_O_2_ (30%; Sigma-Aldrich). Chemiluminescence was monitored with help of the Glomax Multi^+^ combined spectro-fluoro-luminometer (Promega Corporation, Madison, WI, USA). The results are expressed as relative light units/s/g protein [24].

The total antioxidant capacity (TAC) of the lysates was assessed with an improved chemiluminescence assay utilizing a signal reagent composed of 282.2 mmol/L luminol, 41.8 mmol/L 4-iodophenol (Sigma-Aldrich), 12 mol/L H_2_O_2_, and 0.4% (v/v) horseradish peroxidase (Sigma-Aldrich) solution in Stabilzyme Select Stabilizer (SurModics, Eden Prairie, MN, USA) [26]. Trolox (6-hydroxy-2,5,7,8-tetramethylchroman-2-carboxylic acid; 5–100 μmol/L; Sigma-Aldrich) was used as a standard for the assay. The chemiluminescent signal was recorded during 10 consecutive 1-minute long cycles using the Glomax Multi^+^ combined spectro-fluoro-luminometer. The results are expressed as Eq. μmol Trolox/g protein [24,25].

Protein oxidation expressed through the concentration of protein carbonyls was performed with the 2,4-dinitrophenylhydrazine (DNPH) method introduced by Weber et al. [27]. Each lysate was prediluted with distilled water to contain 1 mg protein and pretreated with 1 mL trichloroacetic acid (TCA; 20% w/v; Sigma-Aldrich). Subsequently, 1 mL of each lysate was incubated with 1 mL DNPH (10 mM in 2 N HCl; Sigma-Aldrich) at 37°C for 1 h. Then, 1 mL TCA was added to the mixture, which was then centrifuged (300×*g*, 15 min). The resulting pellets were washed three times with 1 mL of ethanol/ethyl acetate (1/1; v/v; Sigma-Aldrich) and resuspended in 1 mL 6 mol/L guanidine hydrochloride (Sigma-Aldrich) before absorbance measurement at 360 nm with the Cary 60 UV-Vis spectrophotometer (Agilent Technologies, Santa Clara, CA, USA). The amount of protein carbonyls (PCs) was calculated using the molar absorption coefficient of 22,000 1/M.cm and is expressed as nmol PC/mg protein [24].

Malondialdehyde (MDA) as a marker of lipid peroxidation (LPO) was quantified with the thiobarbituric acid reactive substances assay, modified for a 96-well plate. Each lysate was pretreated to 5% sodium dodecyl sulfate (Sigma-Aldrich) and subjected to 0.53% thiobarbituric acid (Sigma-Aldrich) dissolved in 20% acetic acid (Centralchem, Bratislava, Slovakia). The samples were boiled at 100°C for 1 h, then cooled down on the ice for 10 min, and centrifuged at 1,750×*g* for 10 min. The obtained supernatant (150 µL) was transferred to a 96-well plate and subjected to absorbance measurement at 540 nm using the Glomax Multi^+^ combined spectro-fluoro-luminometer [24,25]. The results are expressed as µmol MDA/g protein.

Superoxide dismutase (SOD) activity was quantified with the help of the RANSOD commercial kit (Randox Laboratories, Crumlin, UK), while the RANSEL commercial assay (Randox Laboratories, Crumlin, UK) was used for the assessment of glutathione (GSH) peroxidase (GPx) activity. The lysates were processed according to the instructions of the manufacturer, and activities of both antioxidant enzymes were measured with the Rx Monza semi-automatic analyzer. The results are expressed as IU/g protein [24,26].

To quantify the activity of catalase (CAT), we followed the protocol by Beers and Sizer [28]. Each sample was diluted with 0.05 mol/L PBS (pH 7.0) and subsequently exposed to 0.059 mol/L H_2_O_2_ (30%) in 0.05 mol/L PBS. The decrease of H_2_O_2_ was tracked at 240 nm using the Cary 60 UV-Vis spectrophotometer, and CAT activity was calculated from the initial linear portion of the rate curve. The obtained values are expressed as IU/mg protein [24,25].

Reduced GSH was quantified by the Ellman method [29]. Each sample was pretreated with 10% TCA (Sigma-Aldrich) and 10 mmol/L EDTA in Tris buffer (500 mmol/L, pH 8.2; Sigma-Aldrich) and subjected to 10 mmol/L DTNB (5,50-dithiobis-(2-nitrobenzoic acid); Ellman’s reagent; Sigma-Aldrich) dissolved in 0.1 mmol/L potassium PBS with 5 mmol/L EDTA disodium salt (pH 7.5; Sigma-Aldrich). The resulting colorimetric reaction was observed at 412 nm using the Genesys 10 spectrophotometer (Thermo Fisher Scientific, Waltham, MA, USA). The concentration of GSH in the samples was calculated from a standard curve and is expressed as mg GSH/g protein.

### 2.6 Immunological analysis

Levels of interleukin (IL)-1, IL-6, IL-10, and IL-18 were evaluated using commercially available ELISA kits suitable for rat tissue lysates (#RAB0278, #RAB0312, #RAB0247, and #RAB1147, respectively; Sigma-Aldrich). All assays followed a double-sandwich protocol and were carried out according to the instructions of the manufacturer. IL concentrations were determined with the Glomax plate spectrophotometer (Promega Corporation, Madison, WI, USA) at 450 nm and are expressed as pg/mg protein.

### 2.7 Statistical analysis

Statistical analysis was carried out using the GraphPad Prism program (version 8.4.4 for Mac; GraphPad Software Incorporated, La Jolla, CA, USA). The results are expressed as mean ± standard deviation. First, the data were analyzed using the Shapiro–Wilk normality test taking a normal (Gaussian) distribution into consideration. All data sets passed the test with nonsignificant results at the alpha level of 0.05. Differences between the groups were analyzed using one-way analysis of variance followed by the Tukey multiple comparison test, designed to compare the means of three or more independent samples simultaneously. *P*-values <0.05 were considered statistically significant.

## 3 Results

### 3.1 Testicular histology and morphometry

Results obtained from the histological and morphometrical analyses are presented in Table 1. The testicular weight was decreased in both experimental groups in comparison with the control. A significant reduction of the testicular weight was recorded in the ZN group when compared with the control (*P* < 0.05). We also observed that the testicular weight in the ZO group was higher in comparison with that in the ZN group.

**Figure 2:**
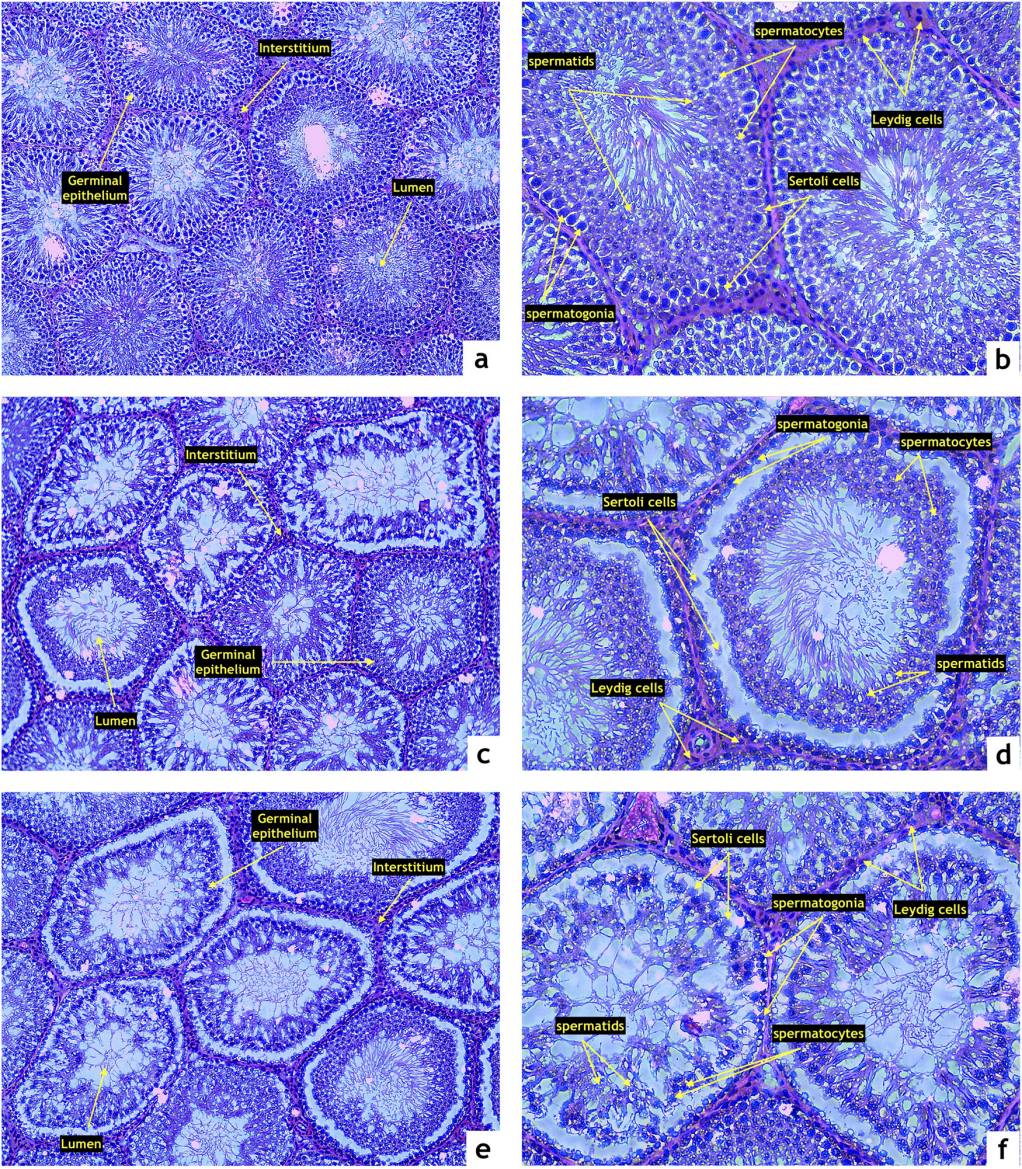
Representative photomicrographs of testicular structure comprising the seminiferous epithelium (SE), lumen (L), and interstitium (IS) stained by hematoxylin and eosin. (a and b) Control (*n* = 15); (c and d) ZDF nonobese (*n* = 15), and (e and f) ZDF obese rats (*n* = 16). Light microscopy; primary magnification 10× (a, c, and e) and 20× (b, d, and f).

Testicular tissue of the control group presented with normal, closely associated seminiferous tubules and intact basal membranes. The spermatogenic line was undisrupted and contained all the layers of spermatogenic cells. The lumen was filled with spermatozoa. No obvious disruptions to the structure or infiltrations to the interstitial space were detected (Figure 2a and b). Testicular structures in the ZN group exhibited irregular basal membranes in numerous seminiferous tubules with disruptions in the seminal epithelium and the spermatogenic series. Atrophic changes were observed in several specimens (Figure 2c and d). Many tubules in the ZO group were structurally damaged with the seminal epithelium being detached from the basal lamina. In numerous cases, the spermatogenic cycle was disrupted with signs of necrosis and denuded spermatogenic cells. The lumen of several seminiferous tubules contained cell debris (Figure 2e and f).

Microscopic observations were furthermore supported by the semi-quantitative analysis of photomicrographs (Table 1). The highest relative volume of the seminal epithelium was found in the control group. Both experimental groups presented a significant decrease in the relative volume of the epithelium in comparison with the control (*P* < 0.05). On the contrary, the highest proportion of the luminar relative volume was found in the ZO group, which was significantly higher when compared with the control (*P* < 0.05).

Assessment of the morphometric characteristics of the seminiferous tubules revealed a continuous decline of the tubular and luminar diameter proportionately to the severity of the metabolic disease. The lowest diameter of the seminiferous tubules as well as the luminar diameter were found in the ZO group, which was significantly different when compared with the control (*P* < 0.05) and the ZN group (*P* < 0.05). A similar phenomenon was observed in the case of the epithelial height, which was significantly lower in the ZN group as well as in the ZO group in comparison to the control (*P* < 0.05; Table 1).

### 3.2 Immunocytochemistry

**Figure 3:**
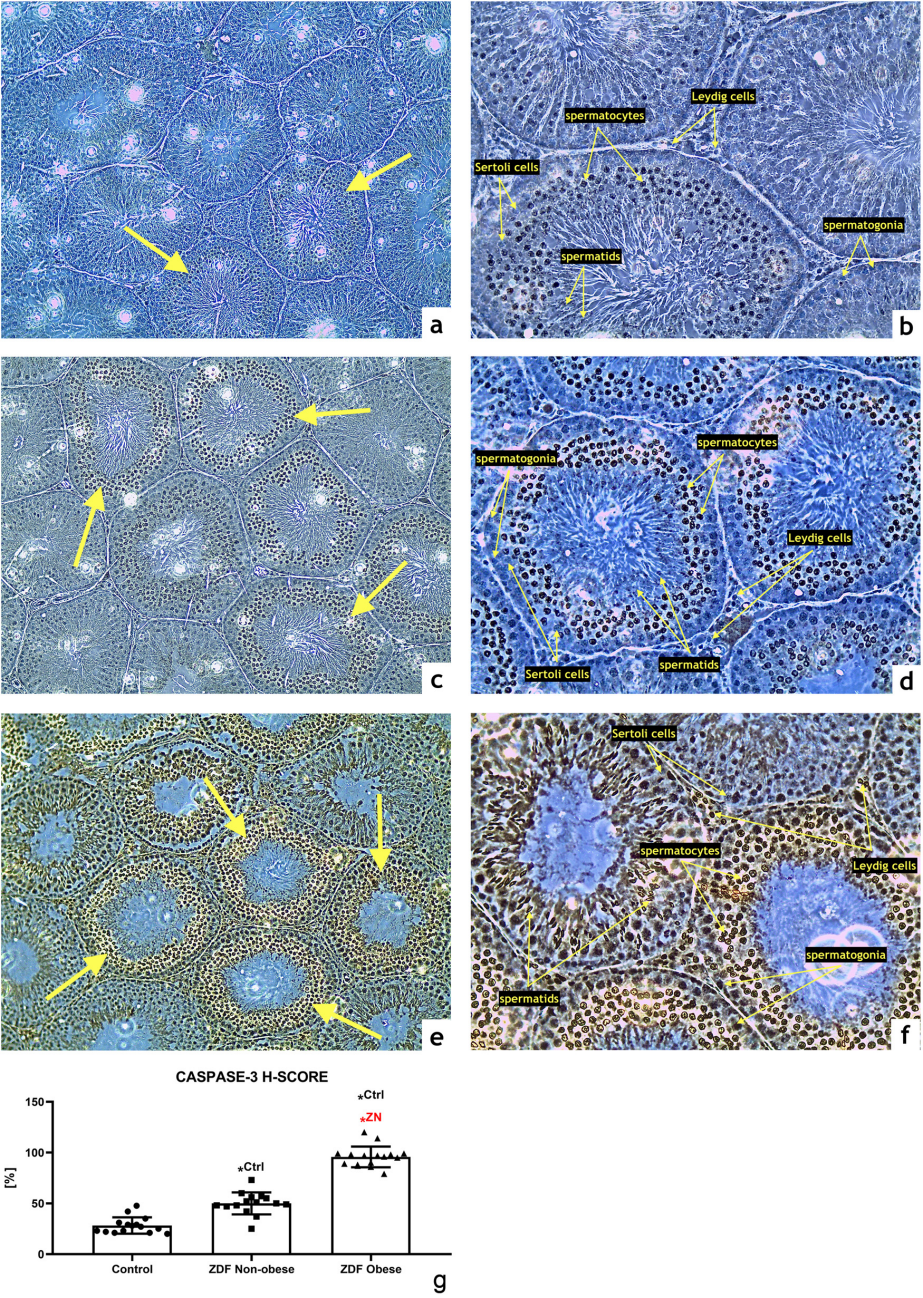
Representative photomicrographs of caspase-3-positive germinal cells of seminiferous tubules in the (a and b) control (*n* = 15); (c and d) ZDF nonobese (*n* = 15) and (e and f) ZDF obese rats (*n* = 16). Light microscopy; primary magnification 10× (a, c, and e) and 20× (b, d, and f). (g) H-SCORE evaluation of the immunocytochemical reaction. Mean ± SD. Significant (^*^) if *P* < 0.05. ^Ctrl^ – versus control; ^ZN^ – versus ZDF nonobese.

Caspase-3 expression was detected particularly within the primary and secondary spermatocytes in all three groups with different intensities. The control group exhibited a weak caspase-3 signal (Figure 3a and b), whereas a moderate expression of the proapoptotic enzyme was observed in the ZN group (Figure 3b and d) with a significantly higher H-SCORE in comparison with the control (*P* < 0.05; Figure 3g). The highest caspase-3 activity was detected in the ZO group with a strong immunocytochemical expression pattern in the whole germline including Sertoli and Leydig cells (Figure 3e and f). The resulting H-SCORE was significantly higher when compared with the control (*P* < 0.05; Figure 3g) as well as the ZN group (*P* < 0.05; Figure 3g).

### 3.3 Oxidative profile

**Figure 4:**
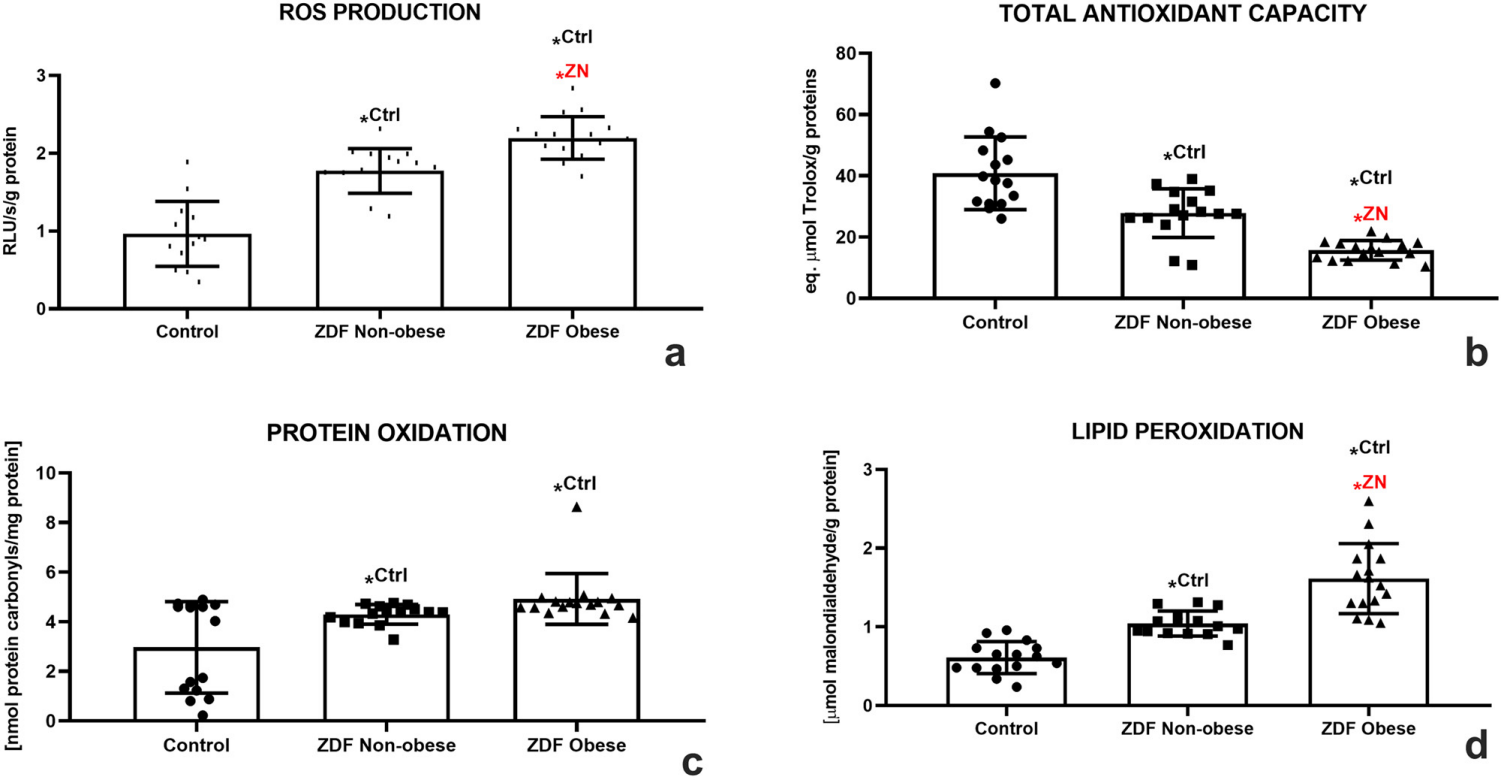
Testicular oxidative profile of the control (Ctrl; *n* = 15); ZDF nonobese (ZN; *n* = 15); and ZDF obese rats (ZO; *n* = 16) expressed through the production of ROS (a), TAC (b), protein oxidation (c), and lipid peroxidation (d). Mean ± SD. Significant (^*^) if *P* < 0.05. ^Ctrl^ – versus control; ^ZN^ – versus ZDF nonobese.

The chemiluminescent analysis revealed a significant increase of ROS in the ZN group in comparison to the control (*P* < 0.05). This process was further aggravated by DM2 and obesity as reflected by significantly higher concentrations of ROS in the ZO group when compared to the control (*P* < 0.05) and the ZN group (*P* < 0.05; Figure 4a), indicating a higher risk for the development of testicular oxidative stress in the animals suffering from chronic hyperglycemia and obesity. A disturbance of the oxidative milieu was corroborated by a significant decline of TAC in both experimental groups when compared with the control (*P* < 0.05 in the case of ZN; *P* < 0.01 with respect to ZO; Figure 4b). The lowest antioxidant strength of the testicular tissue was observed in the ZO group, which was significantly diminished even in comparison with the ZN group (*P* < 0.05).

As unraveled by the DNPH assay, a significantly increased occurrence of protein carbonyls was observed in both experimental groups when compared with the control (*P* < 0.05; Figure 4c). At the same time, DM2 had a profound impact on the extent of testicular LPO, as evidenced by a significant increase in MDA levels in the ZN group when compared to the control (*P* < 0.05). Obesity aggravated the intensity of LPO since the amounts of MDA were significantly higher in the ZO group in comparison with both the control and the ZN group (*P* < 0.05; Figure 4d).

**Figure 5:**
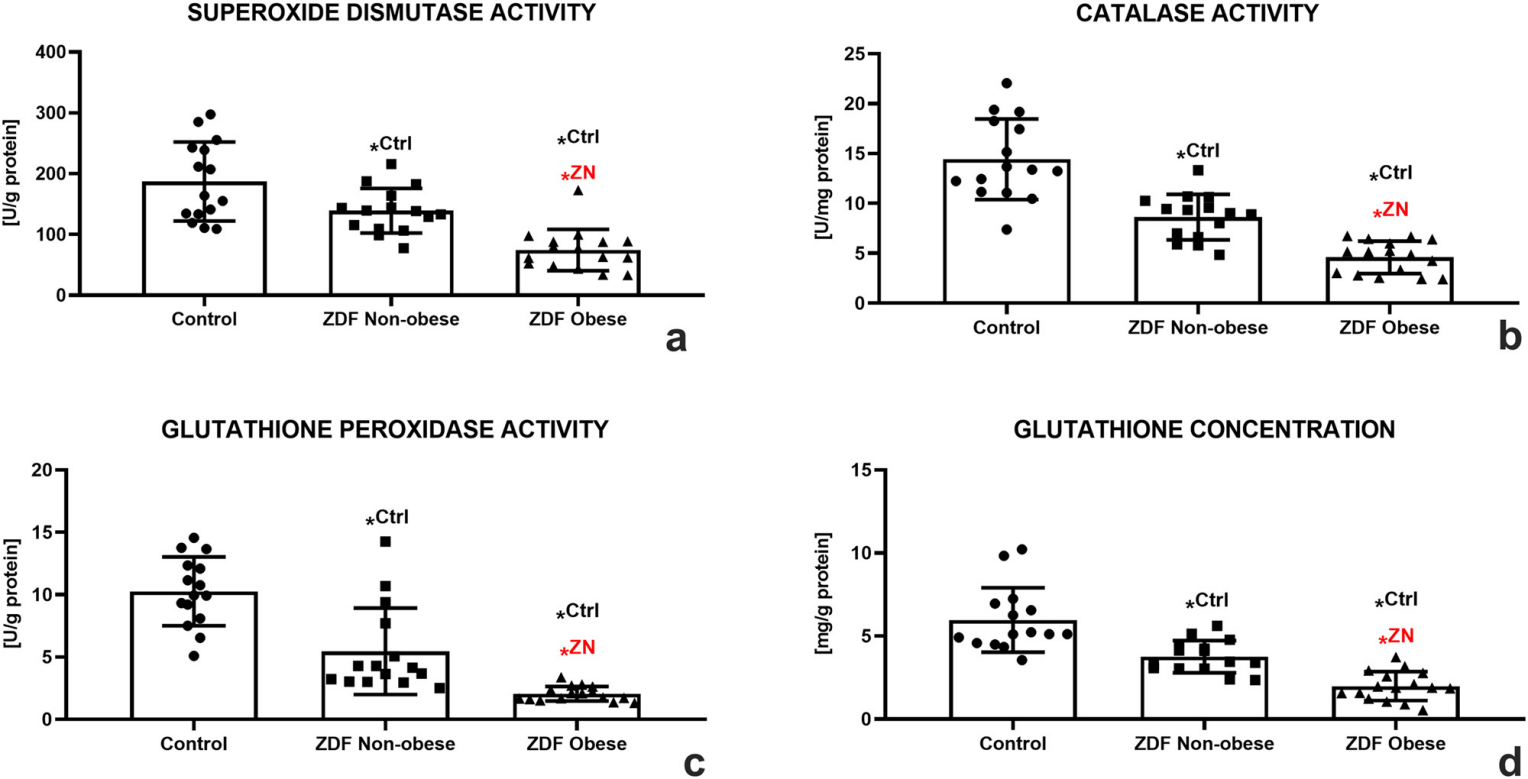
Testicular oxidative profile of the control (Ctrl; *n* = 15); ZDF nonobese (ZN; *n* = 15); and ZDF obese rats (ZO; *n* = 16) expressed through the activity of SOD (a), CAT (b), glutathione peroxidase (GPx) (c), and glutathione (GSH) concentration (d). Mean ± SD. Significant (^*^) if *P* < 0.05. ^Ctrl^ – versus control; ^ZN^ – versus ZDF nonobese.

A deeper look at the principal components of the antioxidant system of male gonads revealed that SOD activity was declining correspondently to the severity of the ailment. A significantly lower SOD activity was detected in the ZN group in comparison with the control (*P* < 0.05). The lowest activity of the enzyme was observed in the ZO group, which was significantly different in comparison to both the control and the ZN group (*P* < 0.05, Figure 5a). CAT seemed to be impacted by both metabolic diseases even more, since a significantly lower activity of this antioxidant enzyme was recorded in both experimental groups as opposed to the control (*P* < 0.05). A significantly different CAT activity was also observed when comparing the ZN and ZO groups (*P* < 0.05; Figure 5b). GPx responded to DM2 or DM2 and obesity in a similar manner as did SOD and CAT. A significantly lower GPx activity was recorded in the ZN group in comparison with the control (*P* < 0.05). Nevertheless, the lowest activity of the enzyme was detected in the ZO experimental group, which was significantly different in comparison with both the control and the ZN group (*P* < 0.05; Figure 5c).

A decreased activity in the prime enzyme of the GSH cycle was well reflected in the dynamics of reduced GSH among the groups distributed in this study. While a decline in the GSH concentration was observed in the ZN group (*P* < 0.05), the lowest amounts of this nonenzymatic antioxidant were detected in the ZO group with significant differences in comparison to both the control and the ZN group (*P* < 0.05; Figure 5d).

### 3.4 Immunological profile

**Figure 6:**
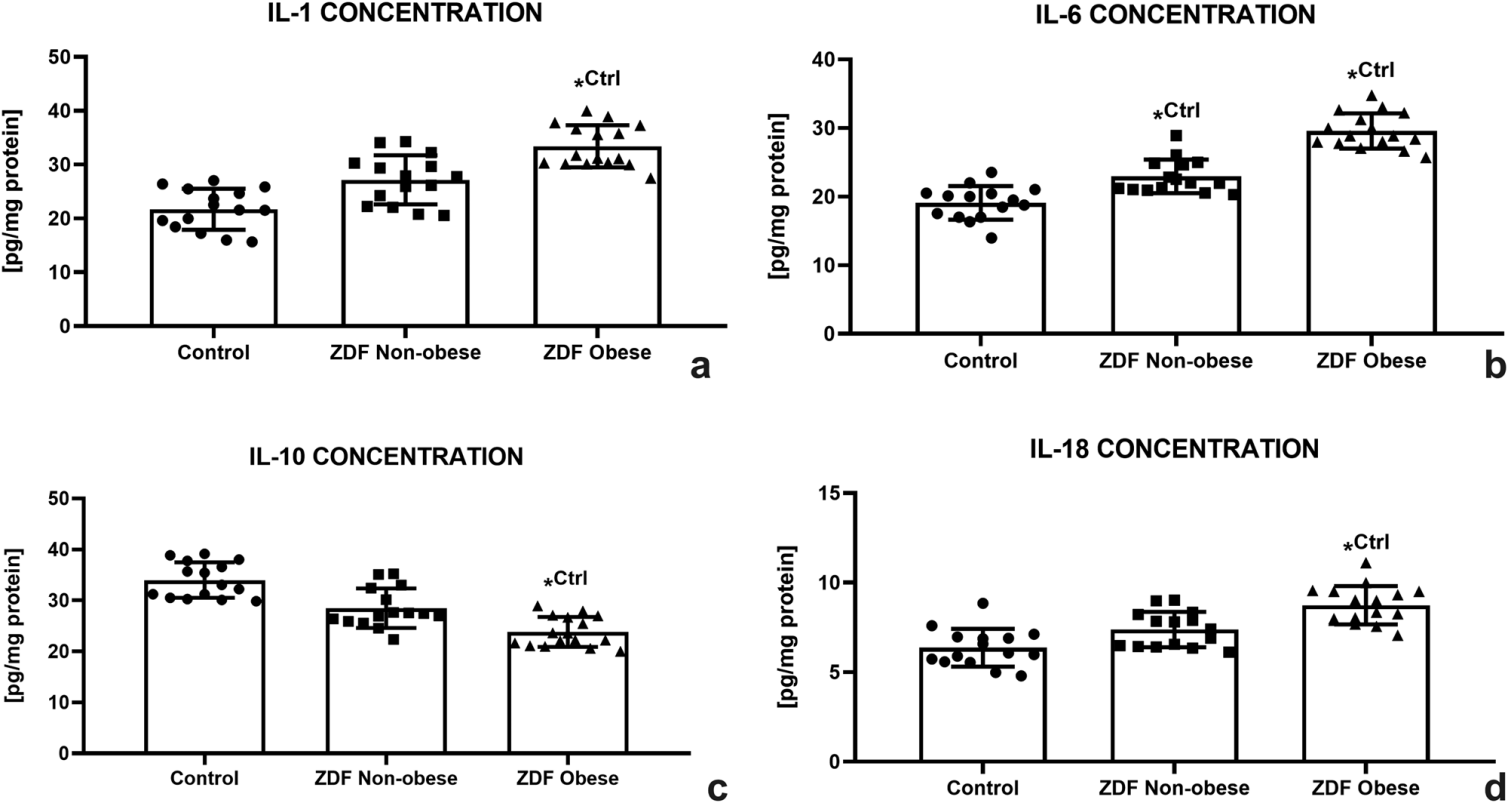
Testicular immunological profile of the control (Ctrl; *n* = 15); ZDF nonobese (ZN; *n* = 15); and ZDF obese rats (ZO; *n* = 16) expressed through the concentration of interleukin-1 (IL-1) (a), interleukin-6 (IL-6) (b), interleukin-10 (IL-10) (c), and interleukin-18 (IL-18) (d). Mean ± SD. Significant (^*^) if *P* < 0.05. ^Ctrl^ – versus control.

As shown in Figure 6a, IL-1 concentration increased in both experimental groups; however, significant differences were observed only between the control and the ZO group (*P* < 0.05). A similar trend was recorded in the case of IL-6; however, significant differences were noted in both experimental groups when compared to the control group (*P* < 0.05; Figure 6b). Inversely, a continuous decrease of IL-10 was observed in the experimental groups. The lowest levels were recorded in the ZO group, which were significantly different in comparison with the control (*P* < 0.05, Figure 6c). Nevertheless, IL-18 revealed an increasing trend corresponding to the health complications of the animals with the highest concentrations detected in the ZO group (*P* < 0.05 in comparison with the control; Figure 6d).

## 4 Discussion

It is well known that diabetes plays a substantial role in testicular pathophysiology through inducing tubular atrophy, damage to the spermatogenic line, and alterations to the blood–testis barrier, which together with chronic inflammation and oxidative stress may contribute to a reduced sperm function [7,30].

A close relationship between DM2 and obesity has been known for decades. Obesity *per se* has a negative impact on male reproduction via endocrine alterations, abnormal sperm function, or changes to the molecular characteristics of the reproductive system. Evidence suggests that impaired spermatogenesis and sexual dysfunction are the primary causes responsible for poor fertility in obese subjects [reviewed by ref. [9]]. Nevertheless, little is known about the impact of the metabolic syndrome encompassing both obesity and DM2 on the testicular architecture and microenvironment. As such, we strived to characterize the structural, oxidative, and immunological properties of testicular tissue collected from ZDF rats suffering from DM2 alone or from DM2 and obesity.

Testicular weight measurements in our study corroborate observations from previous investigations [14,17,31], suggesting that a lower weight of male gonads may have been caused by an ongoing hyperglycemia that leads to the loss of muscle and adipose tissue due to exhaustion of available sources of energy. Conversely, animals suffering from DM2 and obesity displayed higher testicular weight in comparison to ZDF lean rats, most likely due to the presence of excess adipose tissue that seems to compensate for the loss of nourishment for the reproductive cells [32].

Testicular dysfunction is a typical side effect of DM-associated male subfertility. In accordance with our morphometric analysis, previous reports found that the induction of diabetes led to a reduction of the germ cell count that was accompanied by alterations to the spermatogenic series [14,15,33,34]. Correspondingly, DM has been frequently correlated with testicular atrophy and a decline in the proportions of seminiferous tubules, which indicates failure of proper spermatogenesis [16,34,35]. Even though the exact molecular mechanism involved in DM-associated testicular disintegration is not fully understood, it is believed that hyperglycemia or poor glycemic control may be critically involved in this process [36]. Chronic testicular hyperglycemia may lead to impaired communication of the hypothalamic–pituitary–gonadal axis, a disrupted sympathetic innervation and cellular signaling, oxidative stress, increased DNA damage, inhibition of the mitochondrial metabolism, and increased endoplasmic reticulum stress [36,37,38]. Moreover, Maresch et al. [38] suggested that persistently high glucose levels may disrupt prime glucose metabolism pathways, leading to an accumulation of the respective end products within the reproductive tract.

A more severe damage to the testicular tissue was observed in the case of rats suffering from both DM2 and obesity. In accordance with our morphometric data, a meta-analysis by Zhong et al. [39] uncovered reports indicating histopathological alterations in the seminiferous epithelium, accompanied by the presence of cellular debris and germ cell detachment from the basal lamina in testicular tissue from obese subjects. Sertoli cell vacuolization, scattered intraluminal giant cells, and a significant decline of the germinal epithelium thickness were reported as well. On the other hand, Vendramini et al. [14] and Mansour et al. [16] did not observe significant changes in the morphometric properties among ZDF lean and obese rats. This discrepancy may be explained by the age of the animals used in the experiments. While both studies employed pubertal or young adults, we chose to study rats of approximately 9 months of age. The decision to employ older rats was based on selecting a timeframe when complications arising from DM2 were well visible, while the animals were still within their peak reproductive performance [20]. Moreover, as proposed by Salama et al. [17], changes to male reproductive structures caused by DM2 are age dependent and are more pronounced as time progresses.

As previously discussed, several reasons may lay behind a fertility decline in obesity. The endocrine activity of adipocytes may interfere with the hypothalamic–pituitary–testicular axis, affecting the levels of follicle-stimulating hormone, luteinizing hormone, and testosterone [40]. Excessive adipose tissue enclosing the testes may furthermore increase the scrotal temperature leading to a lower testicular weight and sperm quality [41]. Obesity has also been associated with ROS overproduction and DNA fragmentation of the male germ cell line [42]. Finally, global transcriptomic and metabolomic studies uncovered severe alterations in the metabolism of lipids and fatty acids in the testes of ZDF rats, which are essential for normal spermatogenesis and steroidogenesis [18,43].

Our immunocytochemical data suggest that DM2 and/or obesity promote testicular apoptosis in a manner comparable to earlier reports [44,45]. According to Zha et al. [46] and Zhao et al. [47], chronic hyperglycemia alters the testicular proapoptotic Bax/antiapoptotic Bcl-2 ratio. Subsequent upregulation of p53, caspase-8, and caspase-9, indicative of apoptotic signaling, triggers caspase-3. Caspase-3 activation as a major executor of apoptosis recorded in this study was furthermore validated by Nna et al. [48], who observed an upregulation of both mRNA and protein levels of caspase-3 followed by an increased testicular cell apoptosis. Correspondingly, He et al. [45] recorded a notable caspase-3 immunocytochemical signal in spermatogonia, spermatocytes, and Sertoli cells of rats suffering from severe hyperglycemia induced by STZ and high-fat diet.

Oxidative stress, defined as a shift of the prooxidant–antioxidant balance toward ROS production, has been recognized as a key hallmark of hyperglycemia-induced testicular damage [38,49]. Diabetes may be accompanied by ROS overgeneration during the mitochondrial glucose oxidation, which results from high blood sugar levels. Mitochondrial metabolism is sensitive to excessive ROS, and it has been previously established that hyperglycemia represses mitochondrial respiration [5,49]. Excessive amounts of ROS as observed in our study will be subsequently released into the cytoplasm of cells assembling the seminiferous epithelium, outweighing the antioxidant defense system [48,49], and disrupting the spermatogenic process [50]. Testicular germ cells are highly susceptible to oxidative insults because the backbone of their plasma membranes comprises polyunsaturated fatty acids prone to oxidation [51], which may explain a significant increase in LPO recorded in our experimental groups. Furthermore, an increased oxidative pressure on the germ cells may favor an upregulation of caspase-3 and subsequent cell death [49,52,53], which correlates with our immunocytochemical analysis.

Oxidative damage may be even more aggravated if diabetes is associated with obesity, as revealed by our assessment of the testicular oxidative profile. Obesity is defined as an inflammatory condition that is accompanied by a higher metabolic rate and subsequent release of ROS into testicular structures. Pro-inflammatory cytokines may also cause severe damage to the seminiferous epithelium by triggering ROS overgeneration [54,55]. Such activation of the cytokine network will further attract infiltrating phagocytes that are inherently predisposed to release ROS during oxidative outbursts [56].

A disruption of the testicular antioxidant power as observed in the ZN and more so in the ZO group indicates a shift in the oxidative milieu, which may be orchestrated by increased intracellular ROS levels and a higher incidence of oxidative damage to the cells [49,50,57]. Furthermore, this imbalance may be accompanied by a downregulation of critical components of the innate antioxidant capacity, which is consistent with previous studies [44,49,58,59]. The infectivity of SOD to catalyze the dismutation of superoxide to H_2_O_2_, followed by a decreased H_2_O_2_ decomposition to water by CAT and/or the GSH cycle, will lead to its accumulation and subsequent damage to proteins and lipids [60]. As such, our data strongly indicate that the SOD-CAT-GPx antioxidant triangle was significantly impaired in the testes of rats suffering from hyperglycemia, which may have resulted in an increased H_2_O_2_ infiltration to male reproductive tissues.

Diabetes, obesity, and the resulting metabolic syndrome represent a cluster of conditions that may trigger the release of pro-inflammatory biomarkers, which are predictive of insulin resistance [61,62,63]. As suggested by previous studies, chronic hyperglycemia is accompanied by the activation of two major inflammatory pathways, specifically the stress-activated Jun *N*-terminal kinases and the transcription factor nuclear factor-kappa B pathway [64,65]. This inflammatory state accelerated by the release of pro-inflammatory cytokines may be further promoted by the activity of adipokines since these may stimulate additional independent inflammatory responses in obesity [4,66,67]. Among a vast array of molecules considered to act as adipokines, tumor necrosis factor alpha, leptin, adiponectin, ILs, monocyte chemoattractant protein, resistin, and chemokines play major roles in mediating inflammation and causing further disruptions to the glucose homeostasis [55,64,67,68].

In this study, we assessed four major ILs that have been implied in male reproduction. As expected, IL-1 and IL-6 were increasing parallel to the severity of DM and/or obesity in the experimental animals. Accordingly, increased levels of IL-1 have been recorded during a systemic or local inflammation and directly affected testicular steroidogenesis [69]. Similarly, high amounts of IL-6 have been often observed during inflammatory stress that was accompanied by a considerable reduction of testosterone levels [68,70]. In the male gonads, pro-inflammatory cytokines including IL-1 and IL-6 may affect the expression and assembly of the junctional and cytoskeletal proteins, thereby inducing openings of the cell junctions between the adjacent Sertoli and epithelial cells, leading to alterations in the niche of seminiferous epithelium that is essential for spermatogenesis [71,72].

IL-18 is a less known molecule, which may increase particularly during autoimmune pathologies [73] and contribute to the formation of antisperm antibodies that are a common reason for male infertility during systemic inflammation [74]. Elevated concentrations of IL-18 in this study may provide a parallel to caspase-3 expression patterns since it has been suggested that IL-18 is involved in the control of testicular cell proliferation and apoptosis. According to several reports, high IL-18 activity could also inhibit spermatogenesis in sexually mature individuals through enhanced oxygen metabolism [75]. On the other hand, IL-10 represents a major anti-inflammatory cytokine, which was found to be downregulated in patients with impaired fertility. A similar relationship was observed in men with testicular inflammation [76,77], suggesting that IL-10 may inhibit the recognition of antigens by T lymphocytes [78]. In our case, a continuous decrease in this cytokine was observed in both experimental groups, indicating that besides oxidative imbalance, DM2 and/or obesity may disrupt the cytokine network favoring a pro-inflammatory state. Such phenomenon may then cause damage to the testicular tissue and decrease immune protection of male reproductive cells.

Summarizing our experimental data and supporting evidence, we may conclude that the metabolic syndrome associated with DM2 and/or obesity leads to serious alterations in the testicular oxidative profile caused by ROS overproduction, with a concomitant failure of inherent antioxidant mechanisms as well as structural and/or functional damage of proteins and lipids involved in the process of spermatogenesis. Furthermore, chronic inflammation triggered by hyperglycemia, adipokines, and oxidative stress may lead to an imbalance in the immune network, causing an additional risk to the testicular structure and function. All aforementioned phenomena may ultimately result in testicular disintegration and increased germ cell apoptosis, which may contribute to male reproductive dysfunction as a consequence of DM2.

## 5 Conclusion

Our results suggest that the testicular architecture is highly susceptible to pathophysiological changes associated with DM2. Oxidative stress and inflammation may be involved in the progression of testicular dysfunction, which may lead to male subfertility in diabetic and/or obese patients. Finally, we may encourage to use ZDF rats to study therapeutic options in the prevention and/or management of male infertility associated with the progression of DM2.

## References

[1] Tan SY, Mei Wong JL, Sim YJ, Wong SS, Mohamed Elhassan SA, Tan SH, et al. Type 1 and 2 diabetes mellitus: A review on current treatment approach and gene therapy as potential intervention. Diabetes Metab Syndr. 2019;13(1):364–72. 10.1016/j.dsx.2018.10.008 30641727

[2] Banday MZ, Sameer AS, Nissar S. Pathophysiology of diabetes: An overview. Avicenna J Med. 2020;10(4):174–88. 10.4103/ajm.ajm_53_20 PMC7791288 33437689

[3] Reed J, Bain S, Kanamarlapudi V. A Review of current trends with type 2 diabetes epidemiology, aetiology, pathogenesis, treatments and future perspectives. Diabetes Metab Syndr Obes. 2021;14:3567–602. 10.2147/DMSO.S319895 PMC8369920 34413662

[4] Mamdouh M, Shaban S, Ibrahim Abushouk A, Zaki MMM, Ahmed OM, Abdel-Daim MM. Adipokines: potential therapeutic targets for vascular dysfunction in type II diabetes mellitus and obesity. J Diabetes Res. 2017;2017:8095926. 10.1155/2017/8095926 PMC5327767 28286779

[5] Aleissa MS, Alkahtani S, Abd Eldaim MA, Ahmed AM, Bungău SG, Almutairi B, et al. Fucoidan ameliorates oxidative stress, inflammation, DNA damage, and hepatorenal injuries in diabetic rats intoxicated with aflatoxin B1. Oxid Med Cell Longev. 2020;2020:9316751. 10.1155/2020/9316751 PMC7035576 32104544

[6] Chobot A, Górowska-Kowolik K, Sokołowska M, Jarosz-Chobot P. Obesity and diabetes-Not only a simple link between two epidemics. Diabetes Metab Res Rev. 2018;34(7):e3042. 10.1002/dmrr.3042 PMC6220876 29931823

[7] Sonmez A, Yumuk V, Haymana C, Demirci I, Barcin C, Kıyıcı S, et al. Impact of obesity on the metabolic control of type 2 diabetes: Results of the Turkish nationwide survey of glycemic and other metabolic parameters of patients with diabetes mellitus (TEMD obesity study). Obes Facts. 2019;12(2):167–78. 10.1159/000496624 PMC6547285 30893706

[8] Condorelli RA, La Vignera S, Mongioì LM, Alamo A, Calogero AE. Diabetes mellitus and infertility: Different pathophysiological effects in type 1 and type 2 on sperm function. Front Endocrinol (Lausanne). 2018;9:268. 10.3389/fendo.2018.00268 PMC5980990 29887834

[9] Leisegang K, Sengupta P, Agarwal A, Henkel R. Obesity and male infertility: Mechanisms and management. Andrologia. 2021;53(1):e13617. 10.1111/and.13617 32399992

[10] Kottaisamy CPD, Raj DS, Prasanth Kumar V, Sankaran U. Experimental animal models for diabetes and its related complications-a review. Lab Anim Res. 2021;37(1):23. 10.1186/s42826-021-00101-4 PMC8385906 34429169

[11] Capcarova M, Kalafova A. Zucker diabetic fatty rats for research in diabetes. In: Tvrdá E, Yenisetti SC, editors. Animal models in medicine and biology. London: IntechOpen; 2019. p. 75–92.

[12] Nasr NE, Sadek KM. Role and mechanism(s) of incretin-dependent therapies for treating diabetes mellitus. Env Sci Pollut Res Int. 2022;29(13):18408–22. 10.1007/s11356-022-18534-2 35031999

[13] Benko F, Chomová M, Uličná O, Tvrdá E. ZDF Rats: A suitable model to study male reproductive dysfunction in diabetes mellitus type 2 patients. In: Tvrdá E, Yenisetti SC, editors. Animal models in medicine and biology. London: IntechOpen; 2019. p. 93–106.

[14] Vendramini V, Cedenho AP, Miraglia SM, Spaine DM. Reproductive function of the male obese Zucker rats: alteration in sperm production and sperm DNA damage. Reprod Sci. 2014;21(2):221–9. 10.1177/1933719113493511 PMC3879991 23800399

[15] Saito M, Ueno M, Ogino S, Kubo K, Nagata J, Takeuchi M. High dose of Garcinia cambogia is effective in suppressing fat accumulation in developing male Zucker obese rats, but highly toxic to the testis. Food Chem Toxicol. 2005;43(3):411–9. 10.1016/j.fct.2004.11.008 15680676

[16] Mansour M, Coleman E, Dennis J, Akingbemi B, Schwartz D, Braden T, et al. Activation of PPARγ by Rosiglitazone does not negatively impact male sex steroid hormones in diabetic rats. PPAR Res. 2009;2009:101857. 10.1155/2009/101857 PMC2696180 19536350

[17] Salama N, Tsuji M, Tamura M, Kagawa S. Transforming growth factor (beta1) in testes of aged and diabetic rats: correlation with testicular function. Arch Androl. 2001;47(3):217–26. 10.1080/014850101753145933 11695846

[18] Datar J, Regassa A, Kim WK, Taylor CG, Zahradka P, Suh M. Lipid metabolism is closely associated with normal testicular growth based on global transcriptome profiles in normal and underdeveloped testis of obese Zucker (fa/fa) rats. Lipids. 2017;52(11):951–60. 10.1007/s11745-017-4298-2 28965254

[19] Zemancikova A, Torok J, Balis P, Valovic P, Ulicna O, Chomova M. Modulation of sympathoadrenergic contractions by perivascular adipose tissue in mesenteric arteries of rats with different level of body adiposity. J Physiol Pharmacol. 2020;71(4):589–96. 10.26402/jpp.2020.4.14 33316773

[20] Saksena SK, Lau IF, Chang MC. Age dependent changes in the sperm population and fertility in the male rat. Exp Aging Res. 1979;5(4):373–81. 10.1080/03610737908257211 520388

[21] Almášiová V, Holovská K, Andrašková S, Cigánková V, Ševčíková Z, Raček A, et al. Potential influence of prenatal 2.45 GHz radiofrequency electromagnetic field exposure on Wistar albino rat testis. Histol Histopathol. 2021;36(6):685–96. 10.14670/HH-18-331 33779980

[22] Ayan M, Tas U, Sogut E, Caylı S, Kaya H, Esen M, et al. Protective effect of thymoquinone against testicular torsion induced oxidative injury. Andrologia. 2016;48(2):143–51. 10.1111/and.12424 25906970

[23] Kolesarova A, Roychoudhury S, Klinerova B, Packova D, Michalcova K, Halenar M, et al. Dietary bioflavonoid quercetin modulates porcine ovarian granulosa cell functions in vitro. J Env Sci Health B. 2019;54(6):533–7. 10.1080/03601234.2019.1586034 30947605

[24] Kovacik A, Tirpak F, Tomka M, Miskeje M, Tvrda E, Arvay J, et al. Trace elements content in semen and their interactions with sperm quality and RedOx status in freshwater fish Cyprinus carpio: A correlation study. J Trace Elem Med Biol. 2018;50:399–407. 10.1016/j.jtemb.2018.08.005 30262311

[25] Kováčik A, Gašparovič M, Tvrdá E, Tokárová K, Kováčiková E, Rolinec M, et al. Effects of humic acid diet on the serum biochemistery and oxidative status markers in pheasants. Vet Med. 2020;65(6):258–68.

[26] Muller CH, Lee TK, Montaño MA. Improved chemiluminescence assay for measuring antioxidant capacity of seminal plasma. Methods Mol Biol. 2013;927:363–76. 10.1007/978-1-62703-038-0_31 22992928

[27] Weber D, Davies MJ, Grune T. Determination of protein carbonyls in plasma, cell extracts, tissue homogenates, isolated proteins: focus on sample preparation and derivatization conditions. Redox Biol. 2015;5:367–80. 10.1016/j.redox.2015.06.005 PMC4506980 26141921

[28] Beers RF Jr, Sizer IW. A spectrophotometric method for measuring the breakdown of hydrogen peroxide by catalase. J Biol Chem. 1952;195(1):133–40. 14938361

[29] Ellman GL. Tissue sulfhydryl groups. Arch Biochem Biophys. 1959;82(1):70–7. 10.1016/0003-9861(59)90090-6 13650640

[30] Babinets LS, Migenko BO, Borovyk IO, Halabitska IM, Lobanets NV, Onyskiv OO. The role of cytocin imbalance in the development of man infertility. Wiad Lek. 2020;73(3):525–8. 32285827

[31] Srinivasan K, Viswanad B, Asrat L, Kaul CL, Ramarao P. Combination of high-fat diet-fed and low-dose streptozotocin-treated rat: a model for type 2 diabetes and pharmacological screening. Pharmacol Res. 2005;52(4):313–20. 10.1016/j.phrs.2005.05.004 15979893

[32] Fernandez CD, Bellentani FF, Fernandes GS, Perobelli JE, Favareto AP, Nascimento AF, et al. Diet-induced obesity in rats leads to a decrease in sperm motility. Reprod Biol Endocrinol. 2011;9:32. 10.1186/1477-7827-9-32 PMC3068085 21396114

[33] Liu Y, Yang Z, Kong D, Zhang Y, Yu W, Zha W. Metformin ameliorates testicular damage in male mice with streptozotocin-induced type 1 Diabetes through the PK2/PKR pathway. Oxid Med Cell Longev. 2019;2019:1–14. 10.1155/2019/5681701 PMC6906848 31871550

[34] Omar SS, Aly RG, Badae NM. Vitamin E improves testicular damage in streptozocin-induced diabetic rats, via increasing vascular endothelial growth factor and poly(ADP-ribose) polymerase-1. Andrologia. 2018;50(3):1–8. 10.1111/and.12925 29164711

[35] Dkhil MA, Zrieq R, Al-Quraishy S, Abdel Moneim AE. Selenium nanoparticles attenuate oxidative stress and testicular damage in streptozotocin-induced diabetic rats. Molecules. 2016;21(11):1517. 10.3390/molecules21111517 PMC6274080 27869771

[36] Alves MG, Martins AD, Cavaco JE, Socorro S, Oliveira PF. Diabetes, insulin-mediated glucose metabolism and Sertoli/blood-testis barrier function. Tissue Barriers. 2013;1:e23992. 10.4161/tisb.23992 PMC3875609 24665384

[37] Alves MG, Martins AD, Rato L, Moreira PI, Socorro S, Oliveira PF. Molecular mechanisms beyond glucose transport in diabetes-related male infertility. Biochim Biophys Acta. 2013;1832(5):626–35. 10.1016/j.bbadis.2013.01.011 23348098

[38] Maresch CC, Stute DC, Alves MG, Oliveira PF, de Kretser DM, Linn T. Diabetes-induced hyperglycemia impairs male reproductive function: A systematic review. Hum Reprod Update. 2018;24(1):86–105. 10.1093/humupd/dmx033 29136166

[39] Zhong O, Ji L, Wang J, Lei X, Huang H. Association of diabetes and obesity with sperm parameters and testosterone levels: A meta-analysis. Diabetol Metab Syndr. 2021;13(1):109. 10.1186/s13098-021-00728-2 PMC8520257 34656168

[40] Khodamoradi K, Khosravizadeh Z, Seetharam D, Mallepalli S, Farber N, Arora H. The role of leptin and low testosterone in obesity. Int J Impot Res. 2022. 10.1038/s41443-022-00534-y. 35102263

[41] Yaeram J, Setchell BP, Maddocks S. Effect of heat stress on the fertility of male mice in vivo and in vitro. Reprod Fertil Dev. 2006;18(6):647–53. 10.1071/rd05022 16930511

[42] Abbasihormozi SH, Babapour V, Kouhkan A, Niasari Naslji A, Afraz K, Zolfaghary Z, et al. Stress hormone and oxidative stress biomarkers link obesity and diabetes with reduced fertility potential. Cell J. 2019;21(3):307–13. 10.22074/cellj.2019.6339 PMC6582426 31210437

[43] Suh M, Merrells KJ, Dick A, Taylor CG. Testes of obese rats are highly responsive to n-3 long-chain fatty acids. Br J Nutr. 2011;106(7):1005–12. 10.1017/S0007114511001292 21486514

[44] Jiang X, Bai Y, Zhang Z, Xin Y, Cai L. Protection by sulforaphane from type 1 diabetes-induced testicular apoptosis is associated with the up-regulation of Nrf2 expression and function. Toxicol Appl Pharmacol. 2014;279(2):198–210. 10.1016/j.taap.2014.06.009 24967692

[45] He W, Liu H, Hu L, Wang Y, Huang L, Liang A, et al. Icariin improves testicular dysfunction via enhancing proliferation and inhibiting mitochondria-dependent apoptosis pathway in high-fat diet and streptozotocin-induced diabetic rats. Reprod Biol Endocrinol. 2021;19(1):168. 10.1186/s12958-021-00851-9 PMC8576896 34753504

[46] Zha W, Bai Y, Xu L, Liu Y, Yang Z, Gao H, et al. Curcumin attenuates testicular injury in rats with streptozotocin-induced diabetes. Biomed Res Int. 2018;2018:7468019. 10.1155/2018/7468019 PMC6091380 30151389

[47] Zhao Y, Tan Y, Dai J, Li B, Guo L, Cui J, et al. Exacerbation of diabetes-induced testicular apoptosis by zinc deficiency is most likely associated with oxidative stress, p38 MAPK activation, and p53 activation in mice. Toxicol Lett. 2011;200(1–2):100–6. 10.1016/j.toxlet.2010.11.001 21078376

[48] Nna VU, Abu Bakar AB, Ahmad A, Eleazu CO, Mohamed M. Oxidative stress, NF-κB-mediated inflammation and apoptosis in the testes of streptozotocin–induced diabetic rats: Combined protective effects of Malaysian propolis and metformin. Antioxidants. 2019;8(10):465. 10.3390/antiox8100465 PMC6826571 31600920

[49] Sadek KM, Lebda MA, Nasr SM, Shoukry M. Spirulina platensis prevents hyperglycemia in rats by modulating gluconeogenesis and apoptosis via modification of oxidative stress and MAPK-pathways. Biomed Pharmacother. 2017;92:1085–94. 10.1016/j.biopha.2017.06.023 28622709

[50] Nna VU, Bakar ABA, Ahmad A, Mohamed M. Down-regulation of steroidogenesis-related genes and its accompanying fertility decline in streptozotocin-induced diabetic male rats: Ameliorative effect of metformin. Andrology. 2019;7(1):110–23. 10.1111/andr.12567 30515996

[51] Moazamian R, Polhemus A, Connaughton H, Fraser B, Whiting S, Gharagozloo P, et al. Oxidative stress and human spermatozoa: diagnostic and functional significance of aldehydes generated as a result of lipid peroxidation. Mol Hum Reprod. 2015;21(6):502–15. 10.1093/molehr/gav014 25837702

[52] Koh PO. Streptozotocin-induced diabetes increases apoptosis through JNK phosphorylation and Bax activation in rat testes. J Vet Med Sci. 2007;69(9):969–71. 10.1292/jvms.69.969 17917385

[53] Koh PO. Streptozotocin-induced diabetes increases the interaction of Bad/Bcl-XL and decreases the binding of pBad/14-3-3 in rat testis. Life Sci. 2007;81:1079–84. 10.1016/j.lfs.2007.08.017 17870134

[54] Du Plessis SS, Cabler S, McAlister DA, Sabanegh E, Agarwal A. The effect of obesity on sperm disorders and male infertility. Nat Rev Urol. 2010;7(3):153–61. 10.1038/nrurol.2010.6 20157305

[55] Abouzed TK, Sadek KM, Ghazy EW, Abdo W, Kassab MA, Hago S, et al. Black mulberry fruit extract alleviates streptozotocin-induced diabetic nephropathy in rats: targeting TNF-α inflammatory pathway. J Pharm Pharmacol. 2020;72:1615–28. 10.1111/jphp.13338 32754951

[56] Henkel RR. Leukocytes and oxidative stress: dilemma for sperm function and male fertility. Asian J Androl. 2011;13(1):43–52. 10.1038/aja.2010.76 PMC3739401 21076433

[57] Khavarimehr M, Nejati V, Razi M, Najafi G. Ameliorative effect of omega-3 on spermatogenesis, testicular antioxidant status and preimplantation embryo development in streptozotocin-induced diabetes in rats. Int Urol Nephrol. 2017;49(9):1545–60. 10.1007/s11255-017-1636-5 28623529

[58] Artimani T, Amiri I, Soleimani Asl S, Saidijam M, Hasanvand D, Afshar S. Amelioration of diabetes-induced testicular and sperm damage in rats by cerium oxide nanoparticle treatment. Andrologia. 2018;50(9):e13089. 10.1111/and.13089 30022501

[59] Zhao Y, Song W, Wang Z, Wang Z, Jin X, Xu J, et al. Resveratrol attenuates testicular apoptosis in type 1 diabetic mice: Role of Akt-mediated Nrf2 activation and p62-dependent Keap1 degradation. Redox Biol. 2018;14:609–17. 10.1016/j.redox.2017.11.007 PMC5975057 29154192

[60] Aprioku JS. Pharmacology of free radicals and the impact of reactive oxygen species on the testis. J Reprod Infertil. 2013;14:158–72. PMC3911811 24551570

[61] Tsalamandris S, Antonopoulos AS, Oikonomou E, Papamikroulis GA, Vogiatzi G, Papaioannou S, et al. The role of inflammation in diabetes: Current concepts and future perspectives. Eur Cardiol. 2019;14(1):50–9. 10.15420/ecr.2018.33.1 PMC6523054 31131037

[62] Sørgjerd EP. Type 1 diabetes-related autoantibodies in different forms of diabetes. Curr Diabetes Rev. 2019;15(3):199–204. 10.2174/1573399814666180730105351 30058495

[63] Pradhan AD, Manson JE, Rifai N, Buring JE, Ridker PM. C-reactive protein, interleukin 6, and risk of developing type 2 diabetes mellitus. JAMA. 2001;286:327–34. 10.1001/jama.286.3.327 11466099

[64] Shoelson SE, Lee J, Goldfine AB. Inflammation and insulin resistance. J Clin Invest. 2006;116:1793–801. 10.1172/JCI29069 PMC1483173 16823477

[65] Hirosumi J, Tuncman G, Chang L, Görgün CZ, Uysal KT, Maeda K, et al. A central role for JNK in obesity and insulin resistance. Nature. 2002;420(6913):333–6. 10.1038/nature01137 12447443

[66] Frydrych LM, Bian G, O’Lone DE, Ward PA, Delano MJ. Obesity and type 2 diabetes mellitus drive immune dysfunction, infection development, and sepsis mortality. J Leukoc Biol. 2018;104(3):525–34. 10.1002/JLB.5VMR0118-021RR 30066958

[67] Takaoka M, Nagata D, Kihara S, Shimomura I, Kimura Y, Tabata Y, et al. Periadventitial adipose tissue plays a critical role in vascular remodeling. Circ Res. 2009;105(9):906–11. 10.1161/CIRCRESAHA.109.199653 19762682

[68] Sadek KM, Shaheen H. Biochemical efficacy of vitamin D in ameliorating endocrine and metabolic disorders in diabetic rats. Pharm Biol. 2014;52(5):591–6. 10.3109/13880209.2013.854812 24251869

[69] Ganaiem M, AbuElhija M, Lunenfeld E, Cherniy N, Weisze N, Itach SB, et al. Effect of interleukin-1 receptor antagonist gene deletion on male mouse fertility. Endocrinology. 2009;150(1):295–303. 10.1210/en.2008-0848 18787019

[70] Leisegang K, Henkel R. The in vitro modulation of steroidogenesis by inflammatory cytokines and insulin in TM3 Leydig cells. Reprod Biol Endocrinol. 2018;16(1):26. 10.1186/s12958-018-0341-2 PMC5863825 29566712

[71] Chojnacka K, Bilinska B, Mruk DD. Interleukin 1alpha-induced disruption of the Sertoli cell cytoskeleton affects gap junctional communication. Cell Signal. 2016;28:469–80. 10.1016/j.cellsig.2016.02.003 26879129

[72] Zhang H, Yin Y, Wang G, Liu Z, Liu L, Sun F. Interleukin-6 disrupts blood-testis barrier through inhibiting protein degradation or activating phosphorylated ERK in Sertoli cells. Sci Rep. 2014;4:4260. 10.1038/srep04260 PMC3939460 24584780

[73] Boraschi D, Dinarello CA. IL-18 in autoimmunity. Eur Cytokine Netw. 2006;17:224–52. 17353157

[74] Havrylyuk A, Chopyak V, Boyko Y, Kril I, Kurpisz M. Cytokines in the blood and semen of infertile patients. Cent Eur J Immunol. 2015;40(3):337–44. 10.5114/ceji.2015.54596 PMC4655384 26648778

[75] Komsky A, Huleihel M, Ganaiem M, Kasterstein E, Komorovsky D, Bern O, et al. Presence of IL-18 in testicular tissue of fertile and infertile men. Andrologia. 2012;44(1):1–8. 10.1111/j.1439-0272.2010.01090.x 21615452

[76] Camejo MI. Relation between immunosuppressive PGE(2) and IL-10 to pro-inflammatory IL-6 in seminal plasma of infertile and fertile men. Arch Androl. 2003;49:111–6. 10.1080/01485010390129232 12623747

[77] Białas M, Fiszer D, Rozwadowska N, Kosicki W, Jedrzejczak P, Kurpisz M. The role of IL-6, IL-10, TNF-alpha and its receptors TNFR1 and TNFR2 in the local regulatory system of normal and impaired human spermatogenesis. Am J Reprod Immunol. 2009;62(1):51–9. 10.1111/j.1600-0897.2009.00711.x 19527232

[78] Saraiva M, Vieira P, O’Garra A. Biology and therapeutic potential of interleukin-10. J Exp Med. 2020;217(1):e20190418. 10.1084/jem.20190418 PMC7037253 31611251

